# Bullying in school and cyberspace: Associations with depressive symptoms in Swiss and Australian adolescents

**DOI:** 10.1186/1753-2000-4-28

**Published:** 2010-11-23

**Authors:** Sonja Perren, Julian Dooley, Thérèse Shaw, Donna Cross

**Affiliations:** 1 Jacobs Center for Productive Youth Development, University of Zürich, Culmannstrasse 1, 8001 Zürich, Switzerland; 2 Child Health Promotion Research Centre, Edith Cowan University, WA, Australia

## Abstract

**Background:**

Cyber-bullying (i.e., bullying via electronic means) has emerged as a new form of bullying that presents unique challenges to those victimised. Recent studies have demonstrated that there is a significant conceptual and practical overlap between both types of bullying such that most young people who are cyber-bullied also tend to be bullied by more traditional methods. Despite the overlap between traditional and cyber forms of bullying, it remains unclear if being a victim of cyber-bullying has the same negative consequences as being a victim of traditional bullying.

**Method:**

The current study investigated associations between cyber versus traditional bullying and depressive symptoms in 374 and 1320 students from Switzerland and Australia respectively (52% female; Age: M = 13.8, SD = 1.0). All participants completed a bullying questionnaire (assessing perpetration and victimisation of traditional and cyber forms of bullying behaviour) in addition to scales on depressive symptoms.

**Results:**

Across both samples, traditional victims and bully-victims reported more depressive symptoms than bullies and non-involved children. Importantly, victims of cyber-bullying reported significantly higher levels of depressive symptoms, even when controlling for the involvement in traditional bullying/victimisation.

**Conclusions:**

Overall, cyber-victimisation emerged as an additional risk factor for depressive symptoms in adolescents involved in bullying.

## Background

It is well established that students who are bullied by their peers are at higher risk for internalizing problems. Recently, a new form of bullying behaviour has come to the attention of school staff, clinicians, researchers and the general public, namely cyber-bullying. Although several definitions are proposed, cyber-bullying is generally considered to be bullying using technology such as the Internet and mobile phones [1-3]. Recent studies have demonstrated that there is a significant conceptual and practical overlap between both types of bullying such that most young people who are cyber-bullied also tend to be bullied by more traditional methods [4-6]. Despite the overlap between traditional and cyber forms of bullying, it remains unclear if being a victim of cyber-bullying has the same negative consequences as being a victim of traditional bullying. Therefore, to investigate this we differentiate between two types of bullying: *traditional bullying*, including physical or verbal harassment, exclusion, relational aggression and *cyber-bullying*, involving the use of some kind of electronic media (i.e., Internet or mobile phone) to engage in bullying behaviour. The aim of the current study was to investigate the associations between both types of bullying and depressive symptoms in adolescents from two different countries.

### Consequences and correlates of peer victimisation

As children develop, the peer context acquires increasing importance for health and well-being [7]. Peer problems during childhood and adolescence can often result in disruptions to healthy functioning both for those who engage in disruptive behaviours as well as those who are victimised.

It is well established that being a victim of bullying has negative short- and long-term consequences. Furthermore, it is reported that negative peer relations such as lack of acceptance in the peer group and peer victimisation are associated with loneliness, social dissatisfaction and social withdrawal [8] and emotional and behavioural symptoms [9]. Importantly, evidence from several longitudinal studies has demonstrated that peer victimisation and exclusion may also increase children's depressive symptoms [10-13]. These findings indicate that peer rejection and victimisation may play a causal role in the development of depressive symptoms. Consistently, the causal influence of peer victimisation on symptoms of depression was supported by the results of a recent twin study [14].

A meta-analytic review of cross-sectional associations between peer victimisation and psychosocial maladjustment provided clear evidence that peer victimisation is most strongly related to symptoms of depression and least strongly to anxiety [15]. Peer victimisation is also associated with low self-esteem, health problems, suicidality, and poor school adjustment [16-20].

### Consequences and correlates of bullying behaviours

Young people who bully others also often experience negative consequences related to their behaviour, some of which are not immediately apparent [21]. For example, primary and middle school students who bully others often seem unscathed, as their social standing and self-concept are similar to that of observers and markedly better than those who are bullied. Early on, these young people are seen as positive leaders with a good sense of humour, high self-esteem qualities and positive early friendship qualities and popularity [22,23].

Nevertheless, as children grow older bullying behaviours become increasingly maladaptive. Whereas young children solve disputes by fighting, adolescents and adults prefer negotiation to solve a conflict [24]. Children who bully others often do not learn to interact and communicate in socially appropriate ways and therefore have difficulty in interacting adequately with their older peers. This often results in persistent maladaptive behavioural patterns [25], as well as representing an elevated risk for serious injury [26], alcohol dependency [27], and delinquency [28]. These findings suggest that children and adolescents who bully others, frequently also show other forms of antisocial behaviour and that some of those students show a pattern of life-course persistent antisocial behaviour [29].

Furthermore, adolescents who bully others are found to have more psychological and physical problems than their peers [30], and have an increased risk for depression and suicidal ideation [31]. Bullying research traditionally differentiates between children or adolescents who are only victims, only bullies or both [28]. Regarding potential outcomes of bullying, it has been shown that those who both bully others and are victimised (i.e. bully-victims) report the highest levels of externalizing and internalizing symptoms [31,32].

In sum, bullying perpetration and victimisation may have highly negative consequences for children's and adolescents' mental health and well-being. In general, bullying others is most strongly associated with externalizing problems, while being a victim of bullying is strongly associated with internalizing symptoms.

### Consequences and correlates of cyber-bullying and cyber-victimisation

The existing (albeit limited) literature on cyber-bullying suggests that the consequences of cyber-bullying may be similar to traditional bullying. Cyber-bullying, like traditional bullying, correlates significantly with physical and psychological problems [33]. A large scale Australian-based bullying study also demonstrated that cyber-victimisation is associated with higher levels of stress symptoms [4]. Moreover, adolescent victims of cyber-bullying not only reported higher depressive symptoms but also that they engage in other types of problematic behaviour, such as increased alcohol consumption, a tendency to smoke and poor school grades [34]. Cross-sectional studies showed that aggressors are at increased risk for school problems, assaultive behaviours, and substance use [35]. These findings suggest that cyber-victimisation, like traditional victimisation, increases the risk of internalizing (and externalizing) problems.

However, as traditional and cyber-bullying forms are strongly associated and frequently co-occur within the same individuals [1,36-39] it is important to investigate both forms of bullying simultaneously. Few studies have systematically analysed the impact of cyber versus traditional bullying on adolescents' adjustment and mental health.

In a recent study with 761 adolescents from Austria the combined victim group (cyber and traditional victimisation) showed the highest level of internalizing problems [6]. In this study, combined bully-victims showed the most maladjusted pattern. Similarly, a Swedish study found that cyber-victimisation contributed over and above traditional victimisation to adolescents' social anxiety [40]. Cyber-victimisation is also associated with a range of negative emotions [41]. Qualitative data suggest that in comparison with traditional bullying forms, cyber-bullying evoked stronger negative feelings, fear and a clear sense of helplessness [42]. Therefore, being a victim of cyber-bullying might be even more strongly associated with depressive symptoms than traditional victimisation.

### Research questions

This paper describes the relationship between traditional and cyber forms of bullying/victimisation and psychological outcomes. Several hypotheses were generated: (1) there is an overlap between traditional bullying/victimisation and cyber-bullying/victimisation; (2) traditional victims and bully-victims experience higher levels of depressive symptoms than those who bully others and non-involved students; and (3) cyber-victimisation represents an independent risk factor - over and above traditional victimisation - for higher levels of symptoms of depression.

In addition to the three main hypotheses, we examined the influence of culture on the relationship between perpetration/victimisation and outcome. Eslea and colleagues showed in a large dataset from seven different countries that victims of traditional bullying were significantly more disadvantaged on all measures (e.g., mental health, friendships) in all samples, whereas bullies did not differ consistently in all samples. The authors concluded that traditional bullying is a universal phenomenon with many negative correlates for victims and few (if any) for bullies [43]. The consequences associated with cyber-victimisation are not as well established as associations with traditional bullying/victimisation. Moreover, no cross-national comparison has been conducted regarding cyber-bullying so far. Given this, we investigated if the outcomes associated with traditional and cyber forms of bullying were similar for young people in Switzerland and Australia, i.e. we tested whether the results were replicated in both countries (Switzerland versus Australia).

## Method

### Participants

*Australia*. Data for the Australian sample were taken from a cross-sectional study (the Cyber Friendly Schools study) to determine the prevalence of cyber-bullying behaviours in Western Australia (WA) conducted in 2008 by the Child Health Promotion Research Centre (CHPRC) at Edith Cowan University. Schools were randomly selected within strata defined by geographic location and school sector. Non-mainstream and smaller schools as well as those already involved in intervention projects conducted by the CHPRC were excluded, as were students with disabilities which prevented them from completing hard copy self-report surveys. Surveys were administered by school staff within classrooms to those students who consented to participate and for whom written consent was provided by their parents. The Australian students each received a small gift (less than a dollar in value) as thanks for participating in the study. Schools received a $50 voucher for a stationary/educational store and a report detailing study results. All students were provided with contact information for youth support agencies should they have experienced difficulties as a result of participating in the survey. The study was approved by the Edith Cowan University Human Research Ethics Committee.

To increase comparability between the two countries' data and due to different requirements for obtaining consent and subsequent low consent rates in government schools, only results from secondary non-government co-educational schools are reported below.

Relative to the schools included in these analyses, the parent consent rate was 94% with 73% of students returning completed usable questionnaires. Six percent of cases did not indicate gender on the questionnaire and are excluded from the analyses. A total of 22 participants did not indicate their age and those missing values were replaced with the mean age of their respective grade level. This sample comprised 1320 adolescents (Mean age = 13.7, *SD *= 0.92) from four religious-affiliated average socio-economic status schools (two metropolitan, two rural). The final sample was fairly evenly distributed between year levels (Australian Grade 8: 33.8%, Grade 9: 37.2%, Grade 10: 29.0%), by area (48.5% metropolitan) and by gender (52.8% female). Students' access to technology was high: 95% had access to the internet at home and about 92% had their own mobile phone.

*Switzerland*. Nineteen school classes (Grades 7 to 9 in the city of St. Gallen) participated in the study [44]. Schools and participating classrooms were selected to represent all city districts (Schulkreise) and to represent all three school types at the secondary level in Switzerland: *Realschule *with basic classes (low achievement level school, N = 7 classes), *Sekundarschule *with broader classes (average achievement level school, N = 6 classes) and *Kantonschule *with advanced classes (high achievement level school, N = 6 classes).

Following Swiss legislation, permission from the respective school councils to conduct the study was first obtained. Second, teachers from the selected schools volunteered. The survey procedure and the goal of the study were explained to the students who then had the opportunity to refrain from participation without negative consequences (informed oral consent). Students who did not want to participate were offered another activity during the respective school hour. Participating school classes received a voucher for books and media worth 50 Swiss Franks. Teachers and students received general feedback about the occurrence of bully/victim behaviours in their classes and an information flyer that provided contact information for students who may require help following completion of the survey.

Eight students were absent on the day of assessments and did not participate. Although no student actively refused to participate in the study, 6 questionnaires were not included in the study due to missing or incomplete information. The final study sample comprised 374 participants (53.2% female; mean age = 14.3 years, *SD *= 1.13). In total, 17 participants did not indicate their age and these missing values were replaced with the mean age of their respective school class. The sample was fairly evenly distributed between year levels: Swiss Grade 7: 31.8%, Grade 8: 31.8%, Grade 9: 36.6%. Half (51%) of participants reported a foreign-language or migration background, 28% spoke (Swiss) German and at least one other language at home and 23% did not speak (Swiss) German within their families. Students' access to technology was high: 97% had access to the internet at home and about 95% had their own mobile phone.

### Assessment of traditional bullying and victimisation

In the following we differentiate between *bullying *(= perpetration) and *victimisation *(being a victim of bullying).

*Australia*. Participants reported on the frequency of traditional bullying and victimisation in the last 3 months (0 = never to 4 = most days this term). The 6 items address specific negative behaviours (was ignored/excluded; teased in nasty ways; physically hurt; frightened by what someone said they would do; hurtful rumours spread; property stolen, damaged or destroyed).

*Switzerland*. Participants reported on the frequency of traditional bullying and victimisation in the last 3 months (0 = never to 4 = several times a day). The 6 items were used to measure specific negative behaviours (verbal aggression, physical aggression, exclusion, indirect aggression, threat and property-related behaviours).

*Both samples*. Each of the 6 items described above were chosen from a larger item pool of items to make the assessments as similar as possible. Students' self-reports regarding the frequency of being a perpetrator or victim of different forms of traditional bullying were used for categorization into four mutually exclusive categories as bully-victims, victims, bullies, and non-involved students. The same cut-off was used in both samples (at least once a week on at least one item) to denote frequent bullying perpetration/victimisation.

### Assessment of cyber-bullying and -victimisation

*Australia*. The frequency of cyber-bullying and cyber-victimisation were assessed in the same way as described for the traditional bullying (same time period and response options). Each scale encompassed 5 items (sent nasty or threatening emails, nasty messages on the Internet/to mobile phone and mean or nasty comments or pictures sent to websites/other students' mobile phones). Composite scores were calculated for the cyber-bullying behaviours by applying confirmatory factor analysis (see below).

*Switzerland*. Students also reported on the frequency of cyber-bullying and cyber-victimisation (same time period and response options as above). Each scale encompassed 2 items: being bullied through the use of mobile phones (calls, SMS, pictures, films); being bullied through the use of Internet (e-mail, social networking sites, chat). A mean score was computed to establish the scales.

*Both samples*. Due to the nature of cyber-bullying, repetition as a defining feature of this bullying behaviour may be hard to assess [5]. Therefore, no established cut-offs for being a cyber-bully or cyber-victim exist. In addition, dichotomising these scores would have led to an unnecessary loss of information with regard to various degrees of perpetration/victimisation. Thus, cyber-victimisation and cyber-bullying were analysed as linear variables. Whilst the response categories varied between the studies, this was mostly at the upper end of the scale where there were relatively few responses.

### Assessment of depressive symptoms

*Australia*. Students completed a 14-item depression subscale of the Depression Anxiety Stress Scales (DASS) [45].

*Switzerland*. Students completed an 8-item scale addressing depressive symptoms. The scale has been validated in a longitudinal study [46,47].

*Both samples*. Both scales tap the same constructs: sad/depressed feelings, lack of positive feeling, lack of motivation/energy, worthlessness of life. Composite scores were calculated for the depressive symptoms by applying confirmatory factor analysis fitting a single-factor measurement model using weighted least squares estimation based on polychoric correlation matrices. This approach appropriately accounts for the skewed item distributions and measurement error in the items. To maximize data available for analyses, when 20% or less of the items were missing, values were imputed for the missing items based on observed items using the EM (expectation-maximization) algorithm prior to the factor analysis.

### Data analyses

Data analyses accounted for the skew of the dependent variables through the use of tobit regressions, the data were log transformed to meet the requirement of normality of the non-censored scores as recommended by Osgood [48]. Our analyses also accounted for non-independence of the data resulting from the clustered sampling, which can lead to inflated Type I error rates, through the inclusion of a random intercept in the models. Clustering in the Australian data was by school (where secondary students within a year level move between classes for different subjects) and by class in the Swiss sample.

For the statistical analyses, a significance level of p < 0.05 was used.

## Results

### Descriptive statistics

Table 1 shows means and standard deviations of all study variables by sample and gender.

**Table 1:** Descriptive statistics of all study variables

	*Australian sample* *(n = 1259-1307)*		*Swiss sample* *(n = 369-373)*	
	Female	Male	Female	Male
Being a bully-victim ^a^	19 (2.8%)	27 (4.4%)	5 (2.5%)	9 (5.2%)
Being a victim ^a^	66 (9.6%)	55 (9.1%)	22 (11.1%)	24 (13.8%)
Being a bully ^a^	29 (4.2%)	70 (11.5%)	23 (11.6%)	31 (17.8%)
Cyber-bullying (range 0-4)	Mean = .14 SD = .406 Median = 0	Mean = .14 SD = .446 Median = 0	Mean = .03 SD = .152 Median = 0	Mean = .10 SD = .320 Median = 0
Cyber-victimisation (range 0-4)	Mean = .18 SD = .485 Median = 0	Mean = .12 SD = .452 Median = 0	Mean = .08 SD = .218 Median = 0	Mean = .08 SD = .289 Median = 0
Depressive symptoms (range 0-3)	Mean = .34 SD = .630 Median = .05	Mean = .35 SD = .670 Median = .04	Mean = .59 SD = .637 Median = .37	Mean = .34 SD = .449 Median = .13

^a^Numbers (percentages) of students within each country, (traditional bully-victim categories defined according to involvement in bullying behaviours once a week or more often in the past 3 months).

#### Traditional bully/victim categorization

Across both samples, students' self-reported frequency of traditional bullying perpetration/victimisation were used to categorize participants (cut-off: at least once a week): traditional victims (10.0%), bully-victim (3.6%), perpetrators (9.2%), and non-involved (77.2%). In addition, significant gender differences were found with more boys reporting they were frequently perpetrators (12.9%) than girls (5.9%), χ^2 ^= 31.1, *N *= 1666, *p *< .001. When country specific frequencies were examined (Table 1), significantly more Swiss participants reported bullying others than did their Australian counterparts (14.5% versus 7.7%), χ^2 ^= 20.9, *N *= 1666, *p *< .001.

Country and gender differences regarding the other variables are reported in the multivariable analyses below.

### Bivariate associations

Both types of bullying and victimisation were significantly associated with each other (see Table 2 and Table 3). These relationships remained statistically significant (all *p *< .01) when examined by country, with stronger associations observed in the Australian sample. When comparing the traditional bully-victim categories, 41% of (traditional) bullies, 59% of bully-victims, 30% of victims and 16% of non-involved students reported perpetrating cyber-bullying behaviours at least once or twice. Thirty-nine percent of (traditional) victims, 50% of bully-victims, 22% of bullies and 17% of non-involved students were exposed to cyber-bullying behaviours at least once or twice. The association between bullying behaviour and mental health revealed some interesting results with depressive symptoms being most strongly correlated with traditional victimisation (Spearman's rho = .26 Australian sample, rho = .24 Swiss sample) and cyber-victimisation (rho = .22 Australian sample, rho = .12 Swiss sample).

**Table 2:** Bivariate associations between study variables: Complete sample

*Complete sample*	*Age*	*Being a victim*	*Being a bully*	*Cyber-victimisation*	*Cyber-bullying*	*Depressive symptoms*
Gender (female)	.00	-.04	-.13**	.09*	.01	.07**
Age	--	.00	.13**	.02	.14**	.14**
Being a victim		--	.16**	.24**	.18**	.26**
Being a bully			--	.10**	.28**	.12**
Cyber-victimisation				--	.35**	.18**
Cyber-bullying					--	.24**

Note: Spearman's rho calculated for correlations involving cyber-victimization, cyber-bullying and depressive symptoms, Pearson's correlation calculated for all others

*p < .05, **p < .01

**Table 3:** Bivariate associations between study variables: Australian versus Swiss sample

*Australian: Lower diagonal* *Swiss: Upper diagonal*	*Gender (female)*	*Age*	*Being a victim*	*Being a bully*	*Cyber-victimisation*	*Cyber-bullying*	*Depressive symptoms*
Gender	--	.06	-.07	-.12*	.00	-.16**	.24**
Age	-.03	--	-.15**	.07	-.06	.04	.09
Being a victim	-.03	.05	--	.06	.14**	.07	.24**
Being a bully	-.14**	.13**	.20**	--	.00	.19**	.05
Cyber-victimisation	.06*	.08**	.27**	.14**	--	.35**	.12*
Cyber-bullying	.00	.06*	.21**	.32**	.46**	--	.02
Depressive symptoms	-.01	.10	.26**	.11**	.22**	.24**	--

Note: Spearman's rho calculated for correlations involving cyber-victimisation, cyber-bullying and depressive symptoms, Pearson's correlation calculated for all others

*p < .05, **p < .01 two sided tests

### Overlap of bullying/victimisation forms: Multivariable analyses

Next, two tobit regression analyses were conducted to analyse differences between those who use traditional methods to bully, those who are victimised, the combined group (hereafter bully-victims for brevity) and non-involved students in terms of their tendency to cyber-bully others and be cyber-victimised (as log-transformed linear dependent variables). Age and gender and country were entered as control variables. As we were interested in whether country moderates the associations, location (i.e., Switzerland or Australia) was entered as an interaction effect in a first model.

#### Cyber-victimisation

The bully/victim categorization interaction effect with country was found not to be significant (χ^2^[3]= 6.3, p = .098) and was thus dropped from the model The subsequent analysis yielded significant main effects for the bully/victim categorization, gender and country (see Table 4). As is evidenced by the positive sign for the Z statistic, girls reported higher levels of cyber-victimisation than boys (z = 4.75, p < .001). The Australian students reported being more frequently cyber-victimised than the Swiss students (z = 4.46, p < .001). All of the traditional bully/victim behaviour categories differed significantly from each other (see also Table 5). Bully-victims and victims reported higher levels of cyber-victimisation than non-involved students and bullies, of these victims had lower scores on cyber-victimisation than the bully-victims. Students who indicated they bullied others by traditional means reported higher levels of being cyber-victimised than those non-involved in traditional bullying behaviours.

**Table 4:** Results of the tobit regression predicting cyber-victimisation and cyber-bullying

	*Cyber-victimisation*		*Cyber-bullying*		*Depressive symptoms (M1)*		*Depressive symptoms (M2)*	
	Z	Sig	Z	Sig	Z	Sig	Z	Sig
Gender - female	4.75	< .001	1.02	.307	3.14	.002	2.79	.005
Age	1.48	.138	.67	.502	3.58	< .001	3.31	.001
Country - Australia	4.46	< .001	4.11	< .001	-3.46	.001	-4.36	< .001
Trad. bully/victim behaviors								
Bullies vs non-involved	2.50	.012	9.32	< .001	2.47	.014	1.86	.063
Victims vs non-involved	8.31	< .001	4.79	< .001	9.89	< .001	8.38	< .001
Bully-victims vs non-involved	8.96	< .001	10.6	< .001	8.89	< .001	5.60	< .001
Bullies vs victims	-3.83	< .001	3.64	< .001	-5.18	< .001	-4.53	< .001
Bullies vs bully-victims	-5.88	< .001	-3.48	.001	-6.18	< .001	-4.00	< .001
Victims vs bully-victims	-3.02	.002	-6.31	< .001	-2.33	.020	-0.68	.496
Cyber-victimisation							4.83	< .001
Cyber-bullying							1.52	.127

Note: Cyber-victimisation: R^2 ^= 14.0%; Cyber-bullying: R^2 ^= 16.5%; Depressive symptoms (M1): R^2 ^= 12.8%; Depressive symptoms (M2): R^2 ^= 16.1%

**Table 5:** Summary statistics for cyber-victimisation, cyber-bullying and depressive symptoms by traditional bully/victim categorization

	*Cyber-victimisation*		*Cyber-bullying*		*Depressive symptoms*	
***Traditional bully/victim behaviors***	**Mean**	**SD**	**Mean**	**SD**	**Mean**	**SD**
Bully-victims	0.86	1.309	0.86	1.174	1.09	1.040
Victims	0.37	0.716	0.14	0.328	0.79	0.894
Bullies	0.10	0.250	0.37	0.705	0.42	0.647
Non-involved	0.07	0.215	0.06	0.171	0.28	0.507

#### Cyber-bullying others

A non-significant interaction was also found for cyber-bullying between country and bully/victim categorization (χ^2^ [3] = 4.7, p = .192) Further analysis yielded significant effects for the bully/victim categorization (with all comparisons between categories significant) and country (see Table 4). Those who bullied using traditional methods (bullies and bully-victims) reported higher levels of cyber-bullying than those victimised or not involved, with bully-victims reporting higher frequencies than bullies (see also Table 5). Additionally, the Australian students tended to report more frequently engaging in cyber-bullying behaviours than the Swiss students.

### (Cyber)bullying/victimisation and depressive symptoms (multivariable analyses)

To analyse differences between traditional bullies, victims, and bully-victims in relation to depressive symptoms, the same modelling procedure as described above was used. In the first analysis, only traditional bully/victim categorization was used (including a test of the interaction with country) with age and gender entered as control variables. In the second analysis, cyber-bullying and cyber-victimisation (as well as their interactions with country) were entered as additional independent variables.

#### Traditional bullying/victimisation

The analysis found that the effect of bully-victim categorization was not moderated by country (χ^2^ [3] = 6.0, p = .113). The interaction term was dropped from the analyses. However, bully-victim categorization was a significant predictor of depressive symptoms. In addition, significant gender and country effects emerged (see Table 4). Female students reported higher levels of depressive symptoms (z = 3.14, p = .002) whilst the Australian students had lower scores on average than the Swiss (z = -3.46, p = .001). When comparing the traditional bully-victim categories, all were significantly different from each other, with bully-victims having the highest levels of symptoms, followed by victims, then bullies; non-involved students had the least depressive symptoms (see also Table 5).

#### Cyber-bullying/victimisation as additional risk factor

First, the interactions between each of cyber-bullying and cyber-victimisation and country were tested to assess whether their association with depressive symptoms differed in Australia and Switzerland. As neither of these interaction effects reached significance (cyber-victimisation*country: z = .39, p = .697; cyber-bullying*country: z = 1.76, p = .078), they were dropped from the final model. Upon entering cyber-bullying and cyber-victimisation as additional independent variables, the main effects of traditional bully-victim behaviours remained the same (see Table 4), except that the comparison between bullies and non-involved students and the comparison between victims and bully-victims were no longer significant. In addition, cyber-victimisation was a significant predictor of depressive symptoms, the more frequent the victimisation the higher the level of depressive symptoms (z = 4.83, p < .001).

## Discussion

This study examined the relationship between bullying and victimisation and symptoms of depression in adolescents from two different countries, Switzerland and Australia. Particular attention was paid to different forms of bullying behaviour - specifically traditional forms of bullying (including physical or verbal harassment) and cyber-bullying (using the Internet and/or mobile phone). While the association between traditional and cyber forms of bullying is established [49], to date it remains unclear if being cyber-victimised (over and above traditional victimisation) is associated with increased symptom endorsement.

Although in its relative infancy, the emergent research literature describing the outcomes associated with cyber-bullying/cyber-victimisation is largely consistent with the traditional bullying literature illustrating the robust negative relationship between all forms of bullying/victimisation and mental health. However, what has not yet been clearly described is the cumulative effect of being bullied via traditional *and *cyber means on the mental health of young people [6]. Thus, the third aim of this study was to investigate whether in adolescents, cyber-victimisation is an independent predictor of depressive symptoms, after accounting for self-reported traditional bullying victimisation and to determine the influence of study location (i.e., country) on this association.

### Overlap between traditional and cyber-bullying/victimisation

The first hypothesis, which proposed a relationship between traditional and cyber forms of bullying and victimisation, was supported with statistically significant relationships between traditional and cyber forms of bullying perpetration and victimisation in the expected direction. Importantly, significant correlations were found between cyber-victimisation and gender (female), age, traditional bullying perpetration and victimisation. Furthermore, as participants aged, their self-reported bullying perpetration (traditional and cyber) increased, a relationship that remained significant only in the Australian sample when country-specific report was examined. Overall, all associations were stronger in the Australian sample.

These results add to the theoretical [5] and other empirical evidence [1,4,36-39] demonstrating the relationship between traditional and cyber forms of bullying perpetration and victimisation. In accordance with other studies, our findings suggest that traditional and cyber-bullying form part of the same cluster of socially inappropriate behaviours and argue for a behavioural versus technical approach to intervention programs.

### Traditional victimisation and depressive symptoms

It was also hypothesized that those victimised using traditional methods (victims and bully-victims) would endorse more symptoms of depression than those who only reported bullying perpetration. Support for this hypothesis was found demonstrating that students who reported being victimised and bullying others as well as those only victimised were more likely to report depressive symptoms than were those who reported bullying perpetration only. This result was not moderated by country, indicating that the associations were comparable in both countries.

### Cyber-victimisation and depressive symptoms

Finally, it was hypothesized that cyber-victimisation would represent an additional risk factor - independent of traditional victimisation - for the development of symptoms of depression. Strong support was found for the independent association that cyber-victimisation has with symptoms of depression over and above traditional bullying victimisation i.e. cyber-victimisation accounts for a significant amount of the variation in depressive symptoms even after controlling for possible effects of traditional victimisation. Importantly, this association was not moderated by country, which suggests that the relationship is not culturally dependent.

However, several differences between countries were found. For example, while Swiss students were more likely to report bullying others, the Australian students who bully others were more likely to report also using cyber-strategies. Despite these differences, it was demonstrated that cyber-victimisation was a significant predictor of depressive symptoms - a result that was culturally independent. This result suggests an additional negative mental health status associated with being exposed to bullying via technology, over and above that of being victimised by traditional means. Although fewer students reported being cyber-bullied via technology than traditional methods in both countries, clearly the inclusion of technology represents a risk factor for significantly higher rates of internalizing disorders for those victimised using both cyber and traditional methods.

### Practical implications

The implications of these findings are important (e.g., for intervention programs) and demonstrate the scope of negative impact associated with cyber-victimisation. It is suggested that certain features of cyber-bullying (e.g., anonymity of perpetrator, accessibility of victim) present additional and difficult challenges for young people who are victimised [49]. It is often assumed that these challenges could contribute to a worsened mental health state for those victimised and the results of this study provide evidence in support of this.

Furthermore, some of the cyber-bullying strategies employed (e.g., nasty comments on SNS profiles) [4] mean that the audience potentially aware of the harassment is significantly larger. For example, if mean and nasty comments are posted on a SNS profile (social networking sites) or if an embarrassing picture is posted and the victim is identified in the picture by name (i.e., being *tagged*), all people in their network, in addition to other networks, can potentially see that humiliating content. Therefore, strategies against cyber-bullying should also include educating students about privacy settings and safe internet/mobile practices. Given the difficulty in removing comments or pictures from the Internet and the permanence of information shared online, it is not surprising that cyber-victimisation represent an additional and independent risk factor for the development of depressive symptomatology. Further investigation is needed to clarify if specific elements of cyber-victimisation that are associated with poorer mental health outcomes for young people. For example, what is the impact of bullying via social networking sites given comments, pictures, and video can be viewed by a larger network (i.e., more students). Nonetheless, the results of this study raise important questions, as well as concerns, for those young people experiencing mental health issues in addition to bullying via traditional and cyber methods.

### Strengths and Limitations

There were a number of strengths to this study. This was the first study to describe cultural similarities in relation to the impact of cyber-victimisation on depressive symptom endorsement. Despite some cultural differences (e.g., more Australian students reported using multiple strategies to bully (traditional and cyber) compared to Swiss students), the evidence demonstrating the additive effect of cyber-victimisation on mental health is an important result. Furthermore, the (culturally independent) predictive nature of cyber-victimisation on depressive symptoms provides an important insight into the influence of technology on young people.

Overall, there were some limitations with this study. For example, some items that assessed bullying and victimisation were worded differently between the two data collection countries. Moreover, there were certain differences in the wording of response categories and number of items in both samples. Regarding cyber-bullying/victimisation, we found a significant difference between Swiss and Australian students regarding their use of cyberstrategies to bully others (Australians reporting higher levels of cyber-bullying/victimisation). This finding has important methodological implications. Swiss students reported on two rather global items on cyber-bullying, whereas Australian students reported on five different behavioural descriptors of cyber-bullying. This might have lead to an underreporting of cyber-bullying in Swiss students. Studies in traditional bullying research have shown that global items result in lower prevalence rates of bullying than specific behavioural items [50].

Regarding depressive symptoms, it is important to know that although Australian students reported on more items than Swiss students, the same number of symptoms were assessed (i.e. the Australian students reported on two items for each symptom, Swiss students on 1-2 items). Nevertheless, we found a significant country effect on depressive symptoms. We assume that these country differences are mainly due to methodological differences. It is unlikely that the differences are culturally-based given the similarities between Switzerland and Australia in relation to the prevalence of depressive symptomatology [51,52].

There were some sample limitations (Swiss sample comprised students whose teachers volunteered while the Australian sample is comprised of students at religious-affiliated schools only), however, we do not anticipate that the associations examined would differ markedly from those in the general student population. Although there were some differences in sample demographics (e.g. age), these did not have an impact on the relationship between cyber-victimisation and self-reported depressive symptoms. Moreover, samples were highly similar regarding their access to technology. Other limitations concern the nature of the data collected. First, all measures were self-reports. Second, as with all cross-sectional studies the causal direction of the relationships cannot be determined, and thus our focus has been on associations between the variables involved.

## Conclusion

In conclusion, this study provided evidence of a significant association between traditional and cyber forms of bullying behaviours. We demonstrated that, although several cultural differences exist between Swiss and Australian participants in relation to bullying and victimisation, the relationship between cyber-victimisation and increased endorsement of depression symptoms was culturally independent.

## Competing interests

The authors declare that they have no competing interests.

## Authors' contributions

SP and JD were responsible for the conceptual background of the paper, analyzed and interpreted the data and drafted the manuscript. TS analysed and interpreted the data. DC is grant-holder, conceived and directed the Australian study, and was actively involved in writing up the manuscript. All authors read and approved the final manuscript.
